# RNA sequencing-based analysis of the laying hen uterus revealed the novel genes and biological pathways involved in the eggshell biomineralization

**DOI:** 10.1038/s41598-018-35203-y

**Published:** 2018-11-15

**Authors:** Nirvay Sah, Donna Lee Kuehu, Vedbar Singh Khadka, Youping Deng, Karolina Peplowska, Rajesh Jha, Birendra Mishra

**Affiliations:** 1 Department of Human Nutrition Food and Animal Sciences, University of Hawaii at Manoa, Honolulu, HI 96822 USA; 2 Department of Molecular Bioscienes and Bioengineering, University of Hawaii at Manoa, Honolulu, HI 96822 USA; 3 Bioinformatics core, John A. Burns School of Medicine, University of Hawaii at Manoa, Honolulu, HI 96813 USA; 4 University of Hawaii Cancer Center, Honolulu, HI 96813 USA

## Abstract

Eggshell is the outermost calcified covering of an egg that protects it from microbial invasion and physical damage, and is critical for egg quality. However, understanding of the genes/proteins and the biological pathways regulating the eggshell formation is still obscure. We hypothesized that the transcriptomic analysis of the chicken uteri using RNA-sequencing may reveal novel genes and biological pathways involved in the eggshell biomineralization. RNA-sequence analysis using uteri of laying hens at 15–20 h post-ovulation (layers, n = 3) and non-laying (non-layers, n = 3) hens was carried out. About 229 differentially expressed genes (DEGs) were up-regulated in the layers compared to the non-layers. Kyoto Encyclopedia of Genes and Genomes (KEGG) and Ingenuity Pathway Analysis (IPA) revealed more than ten novel genes and biological pathways related to calcium transport and mineralization in the uterus. Based on the enriched pathways and molecular function analysis, 12 DEGs related to eggshell mineralization were further analyzed in the uteri of layers (3 h and 15–20 h post-ovulation), non-layers and molters using qPCR. Expressions of *OC-116* (regulator of mineralization), *OTOP2* (modulator of cellular calcium influx), *CALCB* (intracellular release of Ca-ions), *STC2* (increases alkaline phosphatase activity), and *ATP2C2* (cellular import of Ca-ions) were significantly higher in the uteri of laying hen at 15–20 h post-ovulation. This study identified the involvement of novel genes and their proposed biological pathways in the regulation of eggshell formation.

## Introduction

The oviduct of a laying hen provides a conducive biological environment for the formation and potential fertilization of the egg. The oviduct has five distinct segments with specialized functions; infundibulum (site of fertilization), magnum (deposition of albumen), isthmus (synthesis of eggshell membranes), uterus (calcification of eggshell), and vagina (oviposition). The uterus consists of the glandular and luminal epithelium whose secretions facilitate in eggshell biomineralization. Though the eggshell constitutes only about 10% of the total egg weight, it has a crucial function of providing microbial and physical protection to the egg. It also provides a definite form to the egg, gaseous exchange, and plays a critical role in ensuring food safety and better hatchability.

Breeders have been continuously developing lines of long-life layers to achieve greater egg lay per bird^1^ and some improvements have been achieved for better eggshell strength, however, studies are required to further understand the eggshell fabric and improve the eggshell quality. Irregularities in the formation of eggshell result in misshaped eggs, soft-shelled eggs, weak-shelled eggs, and sometimes other undesirable egg characteristics with calcite nodules and wrinkles. Approximately, 10% of the eggs produced in poultry farms are lost due to breakage of soft eggshells which accounts for huge economic loss to the egg industry^2^. So far, major selection criteria for eggshell quality has been based on qualitative features such as eggshell specific gravity, breaking strength, and shell deformation^3^. However, selection based on quantitative traits with the knowledge of specific genes/proteins regulating the eggshell structure and quality is required to gain improvements in the eggshell quality in subsequent generations of layer-breeding stock^3^.

Eggshell is composed of 95% calcium carbonate which is transported in the form of calcium and bicarbonate ions from the bloodstream to the uterine cells, and ultimately into the uterine fluid in which the egg bathes^4^. The genetic regulation of eggshell formation and biological pathways involved is highly complex and is not completely understood. Previous studies have reported the expression of several genes in the uterus, however, there is little commonality in the reported genes from each study^4–9^. With the use of cutting-edge techniques and more precise time of sampling, each study reported some unpredicted genes and proposed their potential function in the eggshell mineralization. However, most of the earlier studies employed the long-established microarray technique that limits the detection of novel genes. The eggshell mineralization in chicken is the most efficient biological process of calcium mobilization and biomineralization. This premise leads to the belief that several calcium-transporting genes and proteins must be involved, through independent or integrated biological pathways, for eggshell biomineralization. The genes responsible for eggshell calcification are suppressed in hens before laying period and are activated once the hens reach sexual maturity for egg production^8^. The activation of genes involved in the calcification process is time and tissue-specific^4,6^. Therefore, we hypothesized that RNA sequencing (RNA-seq) of a laying versus non-laying hen’s oviduct can reveal novel genes and biological pathways having a significant role in eggshell formation. The objectives of this study were to analyze the transcriptome of the uteri in layers and non-layers for identifying the differentially expressed genes (DEGs) for eggshell formation, and to further confirm their expression in laying hens at the early egg formation period comparing them with molter and non-laying hens.

## Results

### RNA sequence results and identification of differentially expressed genes

**Figure 1:**
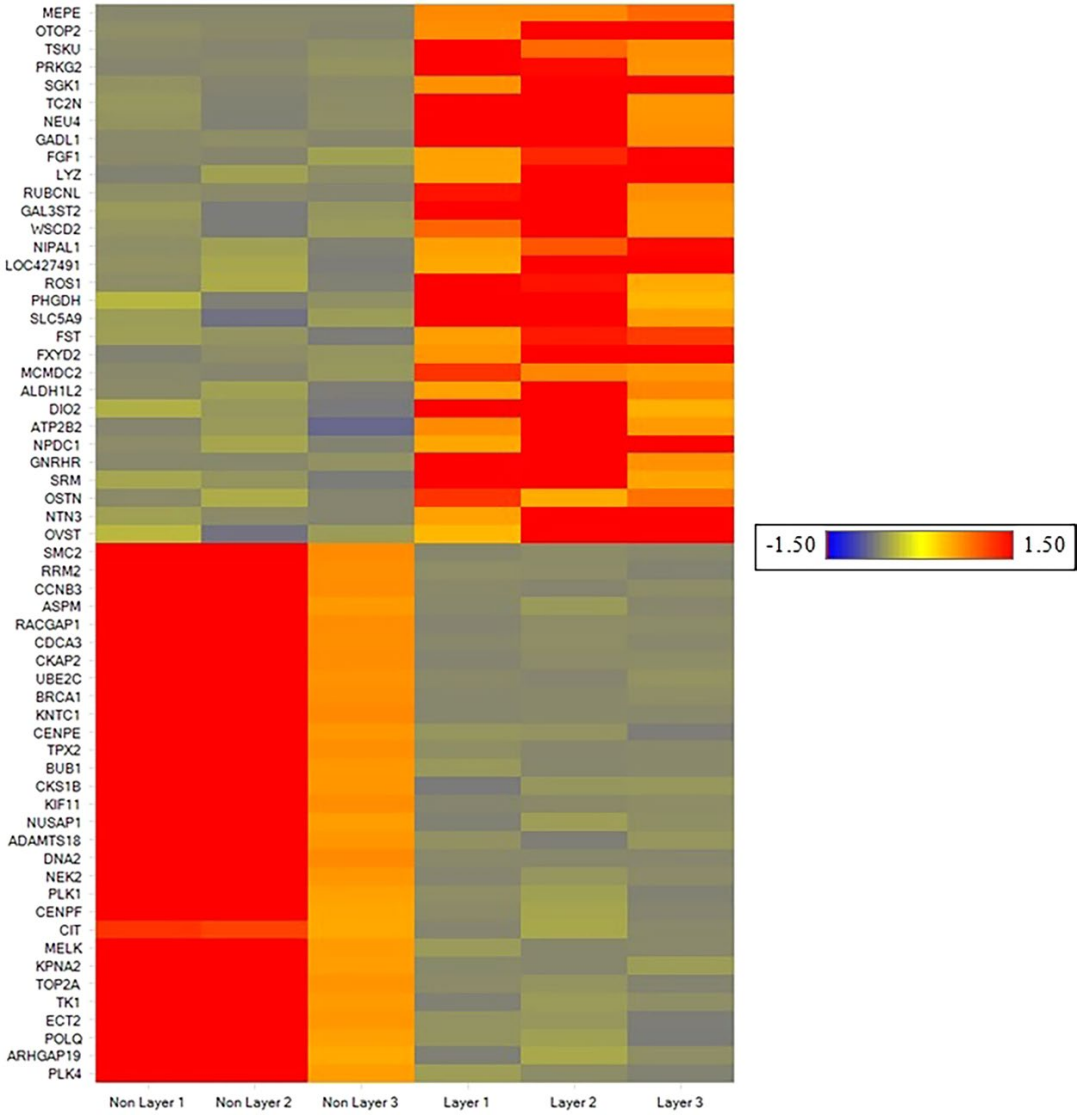
Heat map of thirty up- and thirty down-regulated genes in the uterus of laying and non-laying hens. RNA-seq was performed on three layers (15–20 hours p.o.) and three non-layers’ uteri. Transcripts were aligned to chicken genome and mapped genes with at least 2-fold change difference and Benjamini Hochberg q-value < 0.05 were considered differentially expressed.

Raw reads in FASTQ format from replicated RNA-seq libraries, three each for the layer and non-layer hens, were obtained and their qualities were assessed using FastQC. There were an average of 31.99 M and 28.76 M reads in layers and non-layers, respectively. After trimming and filtration, 96.39% of input reads from layers and 96.55% input reads from non-layers were retained as excellent quality sequences (Supplementary Table S1). An average of 82.96% of the retained reads from layers and 85.12% from non-layers was uniquely mapped to the genome database (Supplementary Table S2). A total of 19,152 gene transcripts were annotated from Ensembl alignment which represents 50.24% of the chicken genome assembly. All the annotated and non-annotated gene transcripts were protein coding. Differential gene expression analysis showed 616 genes were differentially expressed between the layer and non-layer hens (comprehensive gene list in Supplementary Table S3). Among the DEGs, 515 genes were officially characterized while the rest were novel transcripts without any annotation. Of the annotated DEGs, 229 genes were significantly up-regulated and 286 were significantly down-regulated in the laying hens at 15–20 h post ovulation (p.o.) when compared to the non-laying hens. The top 30 over-expressed and under-expressed genes with their relative fold change in laying hens are presented in Table 1 and Table 2, respectively. The heatmap of the 30 most up-regulated and down-regulated genes in layers with respect to non-layers is shown in Fig. 1.

**Table 1:** The 30 most up-regulated differentially expressed genes in the uterus of layers compared to non-layers.

Gene Name	Gene Description	Fold Change
OC-116	ovocleidin-116	174.00
OTOP2	otopetrin 2	15.11
TSKU	tsukushi, small leucine rich proteoglycan	9.85
PRKG2	protein kinase, cGMP-dependent, type II	9.47
SGK1	serum/glucocorticoid regulated kinase 1	8.00
TC2N	tandem C2 domains, nuclear	6.95
NEU4	sialidase 4	6.76
GADL1	glutamate decarboxylase like 1	6.62
FGF1	fibroblast growth factor 1	5.63
LYZ	lysozyme (renal amyloidosis)	5.45
RUBCNL	RUN and cysteine rich domain containing beclin 1 interacting protein like	5.09
GAL3ST2	galactose-3-O-sulfotransferase 2	4.92
WSCD2	WSC domain containing 2	4.76
NIPAL1	NIPA like domain containing 1	4.52
LOC427491	C2 calcium-dependent domain containing 4C-like	4.43
ROS1	ROS proto-oncogene 1, receptor tyrosine kinase	4.29
PHGDH	phosphoglycerate dehydrogenase	4.21
SLC5A9	solute carrier family 5 member 9	4.17
FST	follistatin	4.05
FXYD2	FXYD domain containing ion transport regulator 2	4.01
MCMDC2	minichromosome maintenance domain containing 2	3.97
ALDH1L2	aldehyde dehydrogenase 1 family member L2	3.87
DIO2	deiodinase, iodothyronine type II	3.87
ATP2B2	ATPase plasma membrane Ca2 + transporting 2	3.83
NPDC1	neural proliferation, differentiation and control 1	3.69
GNRHR	gonadotropin-releasing hormone receptor	3.65
SRM	spermidine synthase	3.64
OSTN	osteocrin	3.63
NTN3	netrin 1	3.49
OVST	ovostatin	3.45

Transcripts from uteri of layers and non-layers were aligned to chicken genome and mapped genes with at least 2-fold change difference and Benjamini Hochberg q-value < 0.05 were considered differentially expressed.

**Table 2:** The 30 most down-regulated differentially expressed genes in the uterus of layers compared to non-layers.

Gene Name	Gene Description	Fold Change
SMC2	structural maintenance of chromosomes 2	8.9721
RRM2	ribonucleotide reductase regulatory subunit M2	7.6598
CCNB3	cyclin B3	7.4819
ASPM	abnormal spindle microtubule assembly	6.9292
RACGAP1	Rac GTPase activating protein 1	6.7528
CDCA3	Cell division cycle associated 3	6.5454
CKAP2	cytoskeleton-associated protein 2	6.529
UBE2C	ubiquitin conjugating enzyme E2 U (putative)	6.4307
BRCA1	breast cancer 1	6.3755
KNTC1	kinetochore associated 1	6.2464
CENPE	centromere protein E	6.2078
TPX2	TPX2, microtubule nucleation factor	6.076
BUB1	BUB1 mitotic checkpoint serine/threonine kinase	6.0022
CKS1B	CDC28 protein kinase regulatory subunit 1B	5.5194
KIF11	kinesin family member 11	5.4992
NUSAP1	nucleolar and spindle associated protein 1	5.4481
ADAMTS18	ADAM metallopeptidase with thrombospondin type 1 motif 18	5.4347
DNA2	DNA replication helicase/nuclease 2	5.4128
NEK2	NIMA related kinase 2	5.4027
PLK1	polo like kinase 1	5.2537
CENPF	centromere protein F	5.1474
CIT	citron rho-interacting serine/threonine kinase	5.1143
MELK	maternal embryonic leucine zipper kinase	4.9878
KPNA2	karyopherin subunit alpha 2	4.9424
TOP2A	topoisomerase (DNA) II alpha	4.9072
TK1	thymidine kinase 1	4.8336
ECT2	epithelial cell transforming 2	4.8087
POLQ	DNA polymerase theta	4.7944
ARHGAP19	Rho GTPase activating protein 19	4.7514
PLK4	polo like kinase 4	4.6305

Transcripts from uteri of layers and non-layers were aligned to chicken genome and mapped genes with at least 2-fold change difference and Benjamini Hochberg q-value < 0.05 were considered differentially expressed.

### Functional annotation and pathways enrichment analysis of DEGs

**Figure 2:**
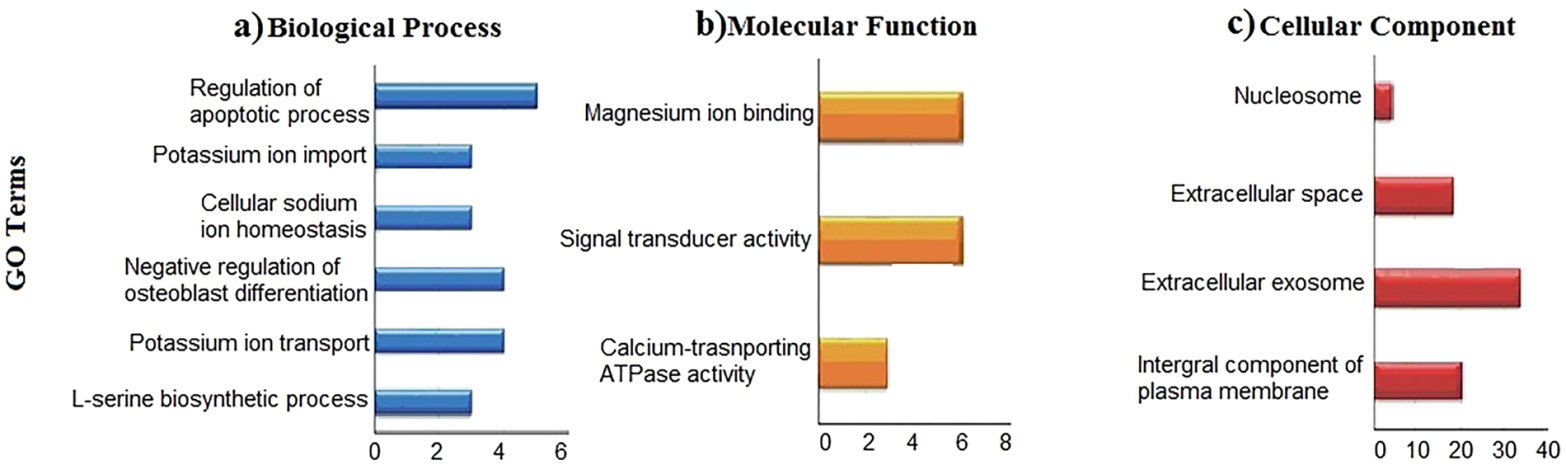
Gene Ontology enrichment analysis of differentially expressed genes in uterus of laying and non-laying hens. (**a**) Biological Process, (**b**) Molecular Function, (**c**) Cellular Component. The 229 up-regulated genes in the layers (15–20 hours p.o.) were subjected to DAVID database for Gene Ontology (GO) enrichment analysis. All the GO terms with a modified Fisher Exact P-value < 0.05 and threshold gene count of 2 were considered enriched.

An open web source, Database for Annotation Visualization and Integrated Discovery (DAVID) system^10^, was used to gain insight into the various Gene Ontology (GO) terms of the upregulated genes in layers. The official gene symbol of the upregulated genes was uploaded to the functional annotation tool in the DAVID system and chicken was selected as the reference genome. Of the 229 genes uploaded, 193 genes were annotated into three GO terms; biological process (BP), cellular component (CC), and molecular function (MF). All the GO terms were considered enriched at a modified P-value < 0.05 and threshold gene count of 2. About 129 genes were enriched for biological process which included serine biosynthetic process, cellular sodium ion homeostasis, and potassium ion transport as the top three over-represented processes in the upregulated set of genes (Fig. 2a). Molecular function had 3 enriched GO terms with 127 genes (Fig. 2b) while cellular component contained 4 enriched GO terms with 143 genes (Fig. 2c). Interestingly, the most enriched GO term for molecular function was calcium-transporting ATPase activity, while signal transducer activity, and magnesium ion binding were the second and third most-enriched. We also analyzed the pathways enrichment for the upregulated genes in layers using Kyoto Encyclopedia of Genes and Genomes (KEGG)^11^. The enrichment parameters were set to a threshold gene count of 2 and a modified Fisher Exact P-value < 0.05. Only 3 pathways were significantly enriched with pantothenate and CoA biosynthesis being the most-enriched followed by calcium signaling pathway, and adrenergic signaling in cardiomyocytes (Table 3).

**Table 3:** KEGG pathway enrichment analysis of upregulated genes in the uterus of layers.

ID	Pathway terms	Fold Enrichment	Genes
gga00770	Pantothenate and CoA biosynthesis	13.6	GADL1, UPB1, VNN1
gga04261	Adrenergic signaling in cardiomyocytes	3.4	ATP2B2, ATP1B1, ADRB1, PLCB4, CREM, ATP1A1
gga04020	Calcium signaling pathway	2.96	ATP2B2, ADRB1, PLCB4, ATP2A3, ADORA2A, P2RX2, AVPR1A

229 up-regulated genes in the layers (15–20 hours p.o.) were subjected to the DAVID database for pathway enrichment analysis. All the pathways with a modified Fisher Exact P-value < 0.05 and threshold gene count of 2 were considered enriched.

### Canonical pathways

**Figure 3:**
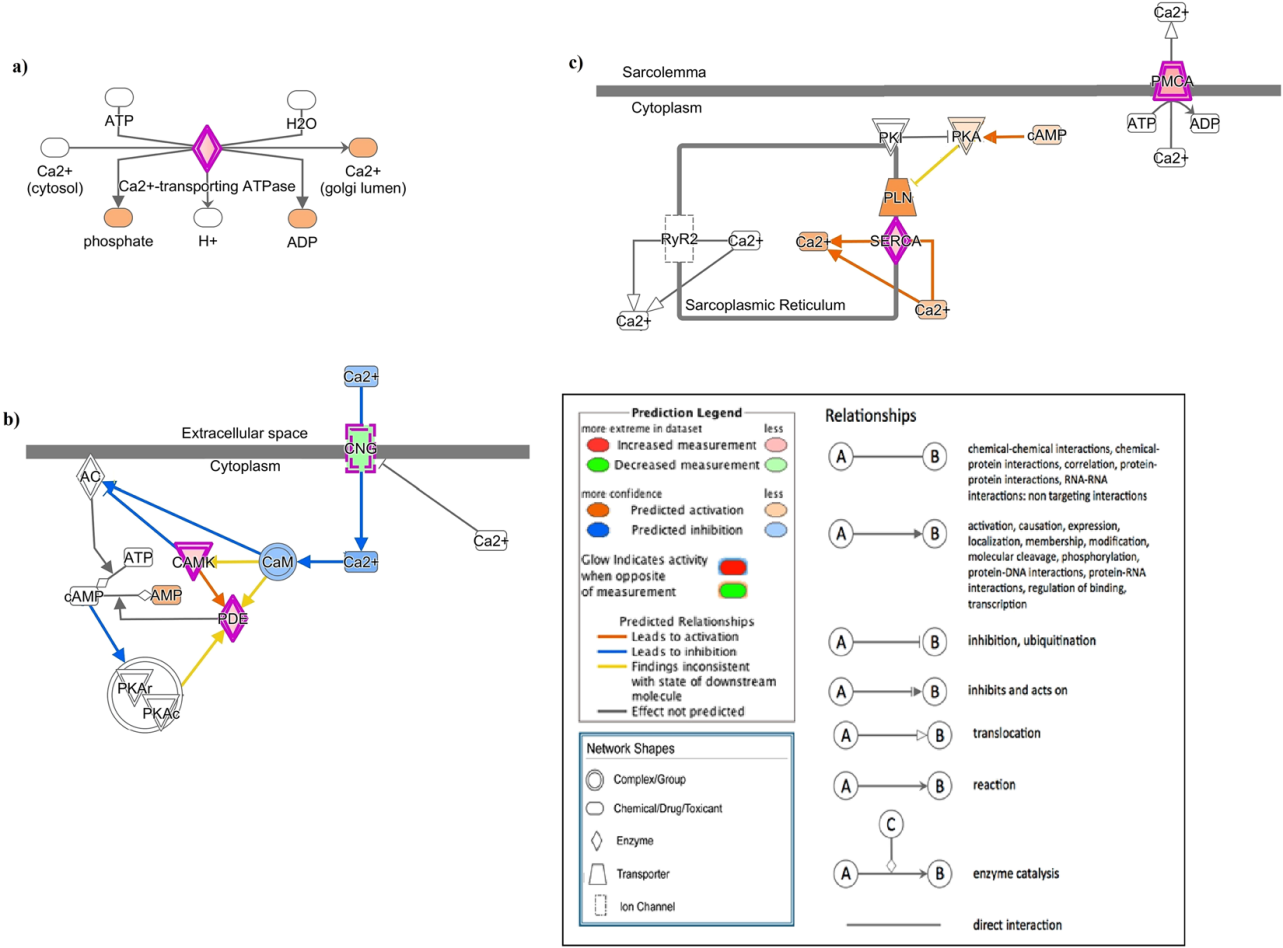
Results of significant canonical pathways associated with ion-transport and the molecules involved. (**a**) Calcium Transport- I pathway. (**b**) cAMP-mediated signaling pathway. (**c**) Cardiac-β-adrenergic signaling pathway. The canonical pathways were analyzed using QIAGEN’s Ingenuity Pathway Analysis (IPA; QIAGEN Inc, https://www.qiagenbioinformatics.com/products/ingenuity-pathway-analysis)^32^. Differentially expressed genes in the layers were subjected to IPA analysis and significant canonical pathways were identified at P-value < 0.05. Above identified canonical pathways demonstrate how the candidate molecules (genes) are involved in ion-transport.

The ingenuity pathway analysis (IPA) recognized 480 molecules of the DEGs in its database and analysis showed that these molecules belonged to 43 significant canonical pathways (Table 4). Cell cycle control of chromosomal replication, cell cycle DNA damage checkpoint regulation, role of BRCA1 in DNA damage response, mitotic roles of polo-like kinase, and estrogen-mediated S-phase entry were the 5 most-significant canonical pathways. Of the 43 significant pathways, 6 pathways (Cell cycle: G2/M DNA damage checkpoint regulation, role of CHK proteins in cell cycle checkpoint control, cell cycle: G1/S checkpoint regulation, cAMP-mediated signaling, p53 signaling, and cardiac B-adrenergic signaling) were predicted to be activated while 9 pathways were predicted to be inhibited; the rest were hard to predict as they had equal weight of evidences on their activation/inhibition state or were hard to predict in general. Serine biosynthesis, and superpathways of serine and glycine biosynthesis I were significant with the greatest proportion of encompassed genes. We were particularly interested in calcium transport I, cAMP-mediated signaling and cardiac β-adrenergic signaling pathways because of their potential activation state, and association in ion-transport (Fig. 3).

**Table 4:** Significant canonical pathways involved in the eggshell formation in layers.

Ingenuity Canonical Pathways	−log(p-value)
Cell Cycle Control of Chromosomal Replication	9.68
Cell Cycle: G2/M DNA Damage Checkpoint Regulation	8.12
Role of BRCA1 in DNA Damage Response	6.73
Mitotic Roles of Polo-Like Kinase	6.7
Estrogen-mediated S-phase Entry	5.94
Cyclins and Cell Cycle Regulation	5.83
Role of CHK Proteins in Cell Cycle Checkpoint Control	5.38
GADD45 Signaling	4.33
DNA damage-induced 14-3-3σ Signaling	4.33
Hereditary Breast Cancer Signaling	4.05
Serine Biosynthesis	3.98
DNA Double-Strand Break Repair by Homologous Recombination	3.7
Antiproliferative Role of TOB in T Cell Signaling	3.63
ATM Signaling	3.49
Superpathway of Serine and Glycine Biosynthesis I	3.45
Cell Cycle: G1/S Checkpoint Regulation	3.2
Breast Cancer Regulation by Stathmin1	3.16
Calcium Transport I	2.94
Cell Cycle Regulation by BTG Family Proteins	2.9
Pancreatic Adenocarcinoma Signaling	2.85
Pyrimidine Deoxyribonucleotides De Novo Biosynthesis I	2.82
Pyridoxal 5′-phosphate Salvage Pathway	2.5
cAMP-mediated signaling	2.31
Gustation Pathway	2.13
Regulation of Cellular Mechanics by Calpain Protease	2.07
p53 Signaling	1.93
tRNA Splicing	1.9
Molecular Mechanisms of Cancer	1.82
Salvage Pathways of Pyrimidine Ribonucleotides	1.72
G-Protein Coupled Receptor Signaling	1.68
Primary Immunodeficiency Signaling	1.67
Dopamine-DARPP32 Feedback in cAMP Signaling	1.52
Oleate Biosynthesis II (Animals)	1.49
Cardiac β-adrenergic Signaling	1.44
Aryl Hydrocarbon Receptor Signaling	1.42
DNA Methylation and Transcriptional Repression Signaling	1.41
Glioma Signaling	1.38
nNOS Signaling in Skeletal Muscle Cells	1.37
Choline Degradation I	1.36
L-DOPA Degradation	1.36
Spermidine Biosynthesis I	1.36
Sulfate Activation for Sulfonation	1.36
Mismatch Repair in Eukaryotes	1.32

All the differentially expressed genes in the layers were used in Ingenuity Pathway Analysis, and significant canonical pathways based on IPA scores were identified.

### Variation in uterine expression of selected genes at different physiological status of hens

**Figure 4:**
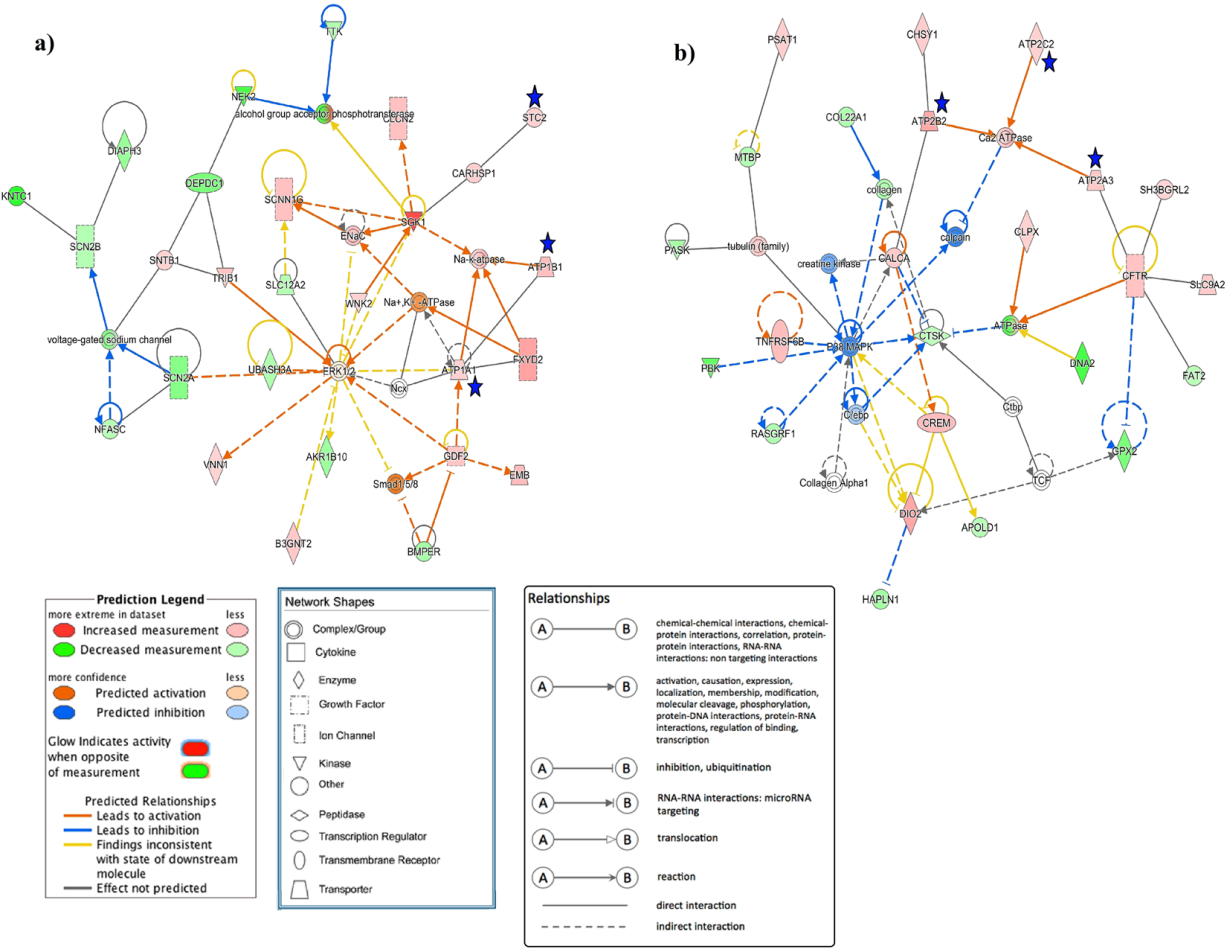
Gene network highlighting the candidate genes and their interaction in potentially regulating the calcium-ion transport during eggshell formation derived from QIAGEN’s Ingenuity Pathway Analysis (IPA; QIAGEN Inc., https://www.qiagenbioinformatics.com/products/ingenuity-pathway-analysis)^35^. (**a**) Gene network for molecular transport, cellular function and maintenance, small molecule biochemistry. (**b**) Gene network for auditory and vestibular system development and function, organismal injury and abnormalities, cancer. Differentially expressed genes in the layers were used in Ingenuity Pathway Analysis and significant gene networks based on IPA scores were identified.

**Figure 5:**
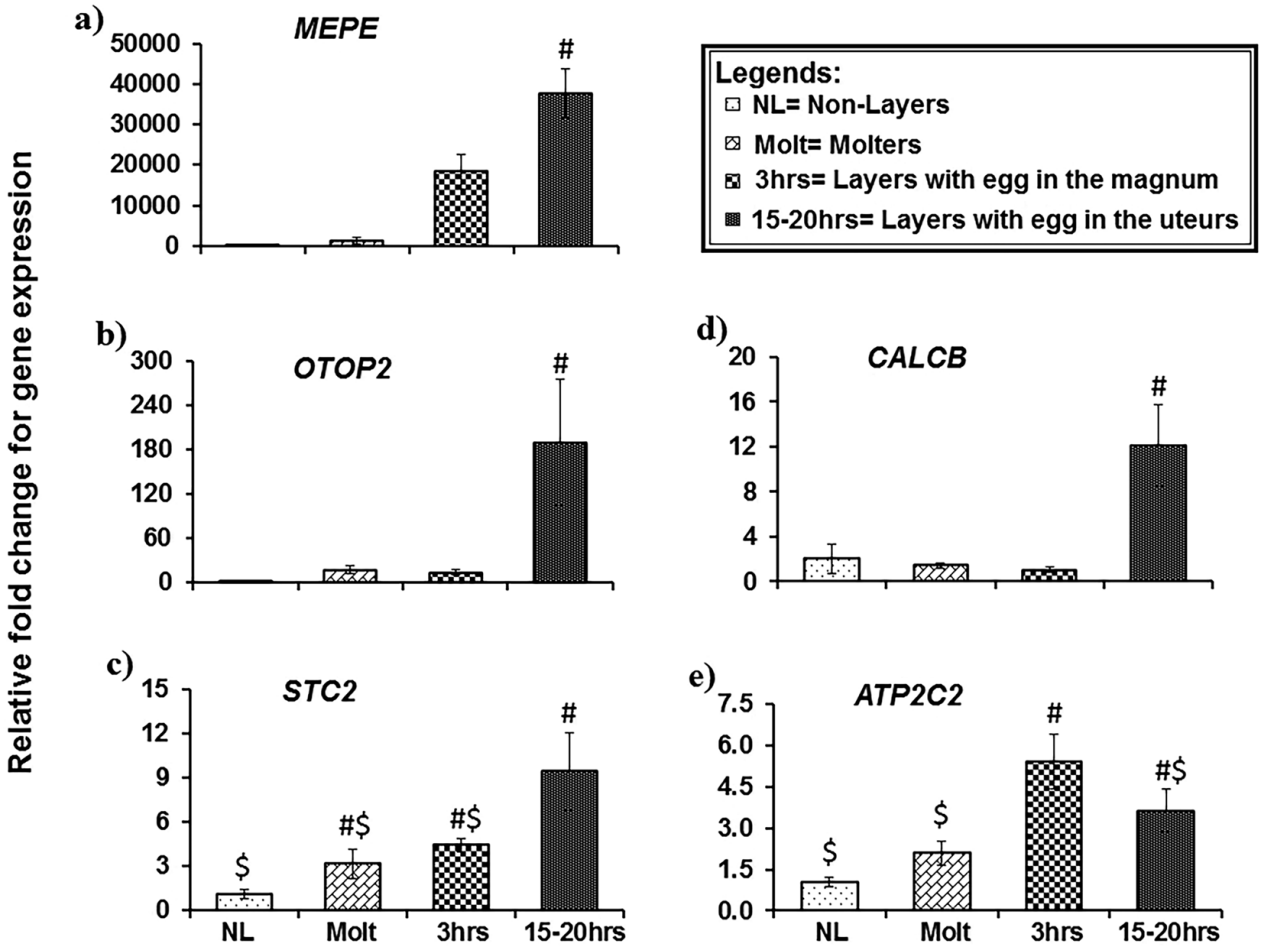
Validation of the gene expression in the uteri of non-laying, molting and laying hens. Data represented as the mean ± SE. X-axis represents different experimental groups; Y-axis represents relative fold change for gene expression. # and $ denotes significance at P-value < 0.05.

The RNA-seq data identified the differentially expressed novel genes in the uteri of laying hens. To determine whether these genes are specific to eggshell calcification, expressions of these genes were further determined in the uteri of laying hens (3 h and 15–20 h p.o.), molting hens and non-laying hens. Twelve candidate genes were selected for validation using qPCR: ovocleidin-116 (OC-116), otopetrin 2 (OTOP2), otopetrin 3 (OTOP3), calcitonin related polypeptide beta (CALCB), stanniocalcin 2 (STC2), osteocrin (OSTN), calcium/calmodulin-dependent protein kinase (CAMK1D), ATPase Na^+^/K^+^ transporting subunit alpha 1 (ATP1A1), ATPase sarcoplasmic/endoplasmic reticulum Ca2^+^ transporting 3 (ATP2A3), ATPase Na^+^/K^+^ transporting subunit beta 1 (ATP1B1), ATPase plasma membrane Ca2^+^ transporting 2 (ATP2B2), and ATPase secretory pathway Ca2^+^ transporting 2 (ATP2C2). The gene network showing the interactions of the identified candidate genes using IPA network analysis is shown in Fig. 4. Among the three house-keeping genes (*TATA-binding protein (TBP), GAPDH*, *B-actin*), expression of *TBP* was most stable in the uterine tissues. So, the relative fold change of the candidate genes was determined using 2^−ΔΔCt^ method after normalization with TBP. Of the 12 candidate genes selected for qPCR validation, five genes showed significant changes between the experimental groups. *OC-116*, *OTOP2*, *STC2* and *CALCB* had the highest expression in hens when the egg was in the uterus (15–20 h p.o.), while *ATP2C2* had the highest expression in hens when the egg was in the magnum (3 h p.o.), followed by intermediate expression intensity in hens with egg in the uterus (Fig. 5). The expression of *OC-116* mRNA in the uterus was 12 thousand-fold higher and 30-fold higher in laying hens at 15–20 h p.o. compared to the non-layers and molters, respectively. Similarly, *OTOP2*, *STC2* and *CALCB* had 71-fold, 8-fold, and 6-fold higher expression levels in the uterus of laying hens at 15–20 h p.o. compared to the non-laying hens, respectively (Fig. 5). In comparison to the molters, the uteri of laying hens at 15–20 h p.o. showed 11-fold, 3-fold, and 8-fold higher expression levels for *OTOP2*, *STC2*, and *CALCB*, respectively. The results of relative fold change for candidate genes obtained from RNA-seq and qPCR were highly correlated (Supplementary Table S4).

## Discussion

Eggshell calcification in the uterus of laying hen involves several cellular and molecular processes in the secretion, transport, and biomineralization of calcium carbonate around the egg.

In this study, we performed gene expression profiling using RNA-seq of uterine samples from laying and non-laying hens. DE-seq analysis between layers’ and non-layers’ uteri revealed several novel genes and biological pathways that potentially regulates the calcium transport and consequently the eggshell formation. Based on the enriched pathways and molecular functions, and their expression levels, twelve genes were identified as potential regulator of calcium-homeostasis during eggshell mineralization. Further, these novel genes were validated in the uteri of laying (3 h p.o. and 15–20 h p.o.), molters and non-laying hens using qPCR. Among the upregulated genes, *OC-116*, *OTOP2*, *STC2*, *CALCB*, and *ATP2C2* were significantly higher in the uteri of laying hens during the eggshell formation (15–20 h p.o.).

Ion-transporting genes/proteins have a significant role in supplying the required amount of calcium and other ions for eggshell mineralization. Previous transcriptomic studies^4–8,11^ in the chicken oviduct have reported that calbindin, ATPases, sodium calcium exchangers, and solute carriers are actively involved in the supply of ions and minerals for eggshell biomineralization. Some well-known genes regulating eggshell mineralization such as calbindin, secreted phosphoprotein, and regulator of calcineurin were detected in our RNA-seq data and we confirmed their differential expression by qPCR (data not shown). However, the scope of this study was to report novel genes that might play complementary role in calcium transport for eggshell biomineralization.

OC-116 also known as MEPE (matrix extracellular phosphoglycoprotein) is an anti-remodeling matrix protein and plays a role in bone- and eggshell- mineralization^4,12,13^. OC-116 is one of the major proteins of the eggshell matrix which is associated with the microvesicular cavities of the eggshell and regulates the calcite crystals organization within the eggshell. OC-116, together with ovocalyxin-32, determines the elasticity, shell thickness, and eventually the shape of the egg^7,14–16^. OC-116 and OC-17 act as framework proteins for calcite crystals deposition during mineralization process^14^. The concentration of OC-17 and some egg-white proteins such as ovotransferrin, ovalbumin, and lysozyme govern the organization of the calcite crystals on the mammillary layer of the eggshell^14,15^. The matrix proteins OC-116 and ovocalyxin-32 then, are involved in the termination of the mineralization process. Both microarray and proteomic studies have confirmed that the expression of OC-116 is dominant at the active calcification stage of mineralization^5,7–9,17,18^. We also observed a very high expression of *OC-116* in the uteri (174-fold by RNA-seq and 12,000-fold by qPCR) during the active eggshell calcification. Similar to our findings, expression of *OC-116* has also been reported by Brionne *et al*.^5^ where comparative expression was determined in laying hens and expelled hens (same as non-laying) that mimics the experimental model of this study confirming the role of OC-116 in eggshell biomineralization.

Calbindin is an intracellular calcium ion transporter and maintains a low concentration of Ca^2+^ in the uterus^4^. In our study, we identified some previously unpredicted otopetrin genes that may participate in the trans-epithelial transport of Ca^2+^ across the uterine plasma membrane into the cytoplasm. The otopetrin gene family has three members; OTOP1, OTOP2, and OTOP3. Though the function of OTOP2 and OTOP3 remains unknown, *Otop1* is postulated to maintain the high concentration of cytosolic calcium in the supporting cells of inner ear during otoconia mineralization in mice^19^. OTOP genes do not necessarily have the same biochemical function as *Otop1*^20^, however, based on their highly conserved nature between vertebrates and a validated up-streamed expression in laying hens during egg calcification, we hypothesized that OTOP2 has a similar role of regulating intracellular calcium in the uterus for eggshell calcification.

Stanniocalcin 2 (STC2) and calcitonin-related polypeptide B (CALCB), also identified for the first time in the uterus, were significantly increased in the laying hens at 15–20 h p.o. STC2 is distributed in a wide variety of tissues and has several functions including osteoblast differentiation^21,22^. It is expressed in chicken joints and its expression is directly related to bone mineralization^22,23^. STC2 acts via activation of extracellular signal-regulated kinase 1/2 (ERK1/2) pathway^22^. The ERK5 pathway, observed in our RNA-seq analysis, has a similar function to the ERK1/2 pathway^24^. CALCB is a member of the calcitonin-related polypeptide family primarily known to regulate cellular calcium homeostasis. It was overexpressed only in the laying hens at 15–20 h p.o. compared to non-layers, molters, and layers at 3 h p.o. suggesting its primary role in eggshell mineralization in hen uteri. CALCB in the uterine epithelium may act through activation of IP3 secondary messenger to cause the intracellular release of free Ca^2+^ ions from the endoplasmic reticulum reserve^25^. Our findings and earlier reports are indicative that STC2 and CALCB are also the novel regulators of mineralization in the eggshell.

The present study also reports the expression of several members of the ATPase family genes in the uterus of laying hens. ATPases are groups of enzymes that help to transport solutes across the cell membrane against their concentration gradient. Three types of ATPases are present in mammals and birds; plasma membrane Ca^2+^ ATPase (PMCAs), secretory pathway Ca^2+^ ATPase (SPCAs), sarcoplasmic reticulum Ca^2+^ ATPase (SERCAs). ATP2B2 is a member of the PMCAs and catalyzes the export of calcium ions in the extracellular space^26^. Based on IPA analysis and canonical pathways, ATP2B2 participates in the calcium signaling pathway which is predicted to be activated during eggshell mineralization. ATP2C2 is a member of the SPCA family of genes. IPA analysis showed that ATP2C2 is a molecule in the significant Calcium Transporting I canonical pathway. It regulates the influx of Ca^2+^ into the cell and the consecutive transport of the cytosolic calcium ions into the Golgi lumen^27^. Expression of ATP2C2 in cancer cells is followed by an increase in luminal Ca^2+^ and initiation of microcalcifications^28^. Based on the functional role of ATP2C2 in different tissues, we speculated that it may participate in eggshell mineralization. We also found ATP2A3 significantly up-regulated in uteri of laying hens. ATP2A3, the member of SERCAs, is an intracellular calcium pump and translocates the cytosolic Ca^2+^ ions to the sarcoplasmic reticulum lumen. Its’ up-regulated status in the uterus suggests that ATP2A3 helps in the act of oviposition.

The top 15 significant canonical pathways belonged to cell cycle, proliferation, and biosynthesis which is reflective of the cellular changes and biological activity occurring in the uterus. However, the focus of this study was on the canonical pathways those are directly related to ion-transport. The DEGs belonged to three significant canonical pathways of interest; (1) Calcium Transport-I, (2) cAMP-mediated signaling, and (3) Cardiac β-adrenergic signaling. Calcium Transport-I pathway constituted of ATP2A3, ATP2B2, and ATP2C2 and involved in the transport of Ca^2+^ ions from cytosol into the golgi lumen (Fig. 3a). Calcium/calmodulin-dependent protein kinase ID (CAMK1D) was one of the molecules that act through the cAMP-mediated signaling pathway. Cyclic nucleotide gated channel alpha 4 (CNGA4), a down-regulated molecule in layers observed in our RNA-seq, was also involved in the cAMP-mediated signaling. CNGA4 has Ca^2+^ mediated negative feedback on the CAMK1D activation (ref.^29^; Fig. 3b). The SERCAs act via the Cardiac β-adrenergic signaling pathways (Fig. 3c). The three canonical pathways associated with calcium-ion remodeling were governed by the ATPases. However, the IPA canonical pathways analysis failed to incorporate the other novel molecules such as OTOP2, STC2, and CALCB. This might be due to the insufficiency of the regulatory and mechanistic information of such novel genes in the uterus.

**Figure 6:**
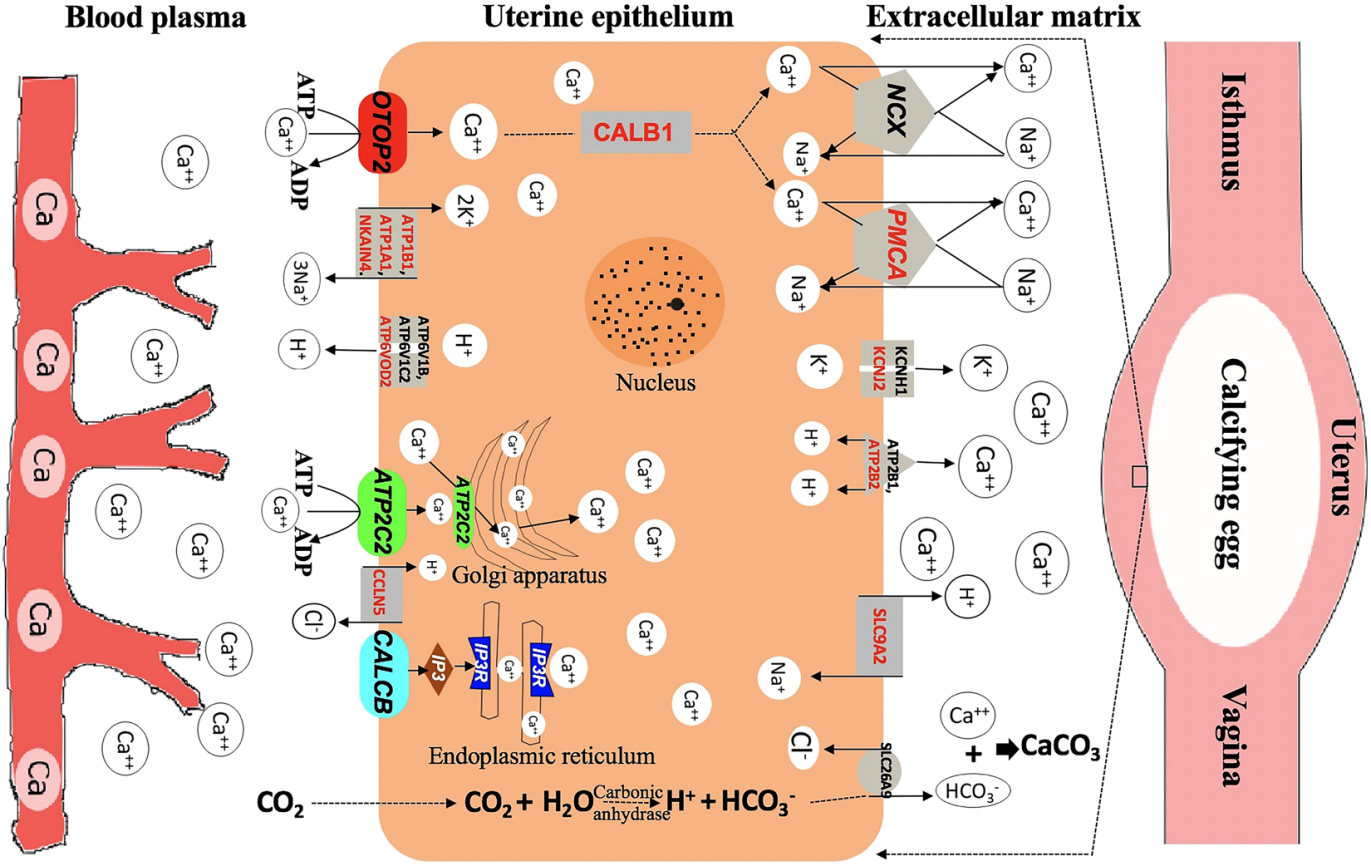
The proposed hypothetical model showing the identified genes and the cognate biological pathways involved in calcium transport for eggshell biomineralization in the uterus. The molecules with colored shapes and red-texts are novel genes detected in this study, whereas, those with grey texts and/or shape are genes described by previous studies^4,5,36^.

The eggshell biomineralization in the hen uteri is a complex process involving the interaction of organic matrix and inorganic solutes to form a hard-calcified membrane that protects the inner content of the egg. With the development of transcriptomic methods, new genes associated with the biological process are being discovered. Using Next Generation Sequencing analysis of uteri, we reported the involvement of eleven novel genes in eggshell mineralization. We further confirmed that *Stanniocalcin 2*, *Otopetrin 2*, *Calcitonin-related polypeptide B* and *ATPase secretory pathway Ca2*^+^ *transporting 2* are the novel genes in the laying hen uterus that are associated with calcium-ion transport and thus, are essential molecules in the eggshell formation. Also, the canonical pathways such as calcium transport I, cAMP-mediated signaling, and cardiac β-adrenergic signaling were identified to be associated with ion-transport during eggshell formation (Fig. 6).

Based on the functional analysis of the novel genes identified in our study as well as previously reported^4,5^, we proposed a hypothetical model showing the identified genes and the cognate biological pathways involved in calcium transport for eggshell mineralization in the uterus (Fig. 6). The massive amount of Ca^2+^ ions required for eggshell mineralization is not reserved in the uterus but continuously supplied through the bloodstream^5^. Under the influence of estrogen, calcium stored in the medullary bones are mobilized into the bloodstream^30^. From the blood capillaries, Ca^2+^ ions move into the extracellular space and are transported in the uterine epithelium by passive transport towards lower concentration gradient maintained by calbindin 1^4^. Several molecules such as ATP2B1 and ATP2B2 directly transport Ca-ions; ATP1A1, ATP1B1, NKAIN4 participate in Na^+^/K^+^ exchange across the cell; and KCNH1 regulates the efflux of K^+^ ions across the uterine epithelium^5^. Besides those reported genes, our findings suggest that Ca^2+^ ions are also transported actively across the uterine epithelium by some novel transporters such as ATP2C2, CALCB and OTOP. The free Ca^2+^ ions influx from the circulation into the uterine epithelial cells further induces the release of Ca^2+^ ions from the intracellular calcium reserves (endoplasmic reticulum and Golgi apparatus)^31^. This huge amounts of intracellular Ca^2+^ ions are then carried by sodium-calcium exchangers (NCX), and plasma membrane Ca^2+^ ATPase (PMCA) into the extracellular matrix where they are combined with bicarbonate ions to initiate mineralization of the eggshell in the uterine lumen^7^. In conclusion, our study provides the expression of several novel genes and biological pathways involved in the Ca^2+^ transportation in the uterus of laying hen and their potential functions in the eggshell biomineralization.

## Materials and Methods

### Animals and husbandry practices

All animal experimentations were carried out in accordance with the guidelines approved by the Institutional Animal Care and Use Committee of the University of Hawaii at Manoa. The husbandry practices, and tissue collection procedures were followed as per standard protocol of Small Animal Facility of College of Tropical Agriculture and Human Resources, University of Hawaii at Manoa. Hy-Line hens were brought from a commercial layer farm in Hawaii and acclimatized for two weeks in the research facility. All hens were housed individually in pens with deep-litter housing under the light regimen of 16 h of light (from 5 am to 9 pm) and 8 h of dark (from 9 pm to 5 am) with *ad libitum* access to feed and water. Hens were monitored daily, and their egg-laying times were recorded. Laying hens (35 weeks old; n = 12) with more than 80% production rate, molters (around 60 weeks old; n = 6), and non-laying hens (35–60 weeks old; n = 6) were used for sampling.

### Tissue collection

Laying history and abdominal palpation were used to presume the presence of egg in the uterus, followed by post-mortem confirmation to determine the total time spent by the egg in the oviduct post-ovulation (p.o.). Molting hens were confirmed based on the laying history and the presence of unovulated eggs in the ovary. Non-laying hens were confirmed based on the absence of any growing follicles in the ovaries. Uteri were collected only from those laying hens where the egg was at albumen deposition stage and at an active calcification stage, i.e., around 3 hours and 15–20 hours p.o., respectively. The eggshell biomineralization starts when the egg is in the uterus and concurrently the expression of the genes involved is supposed to be higher during this period (15–20 h p.o.). From the molting and non-laying hens, uterine tissues were collected at similar time-points parallel to sampling of laying hens. For RNA analysis, portions of the uterine tissues were collected and snapped frozen immediately in liquid nitrogen then stored at −80 °C until analysis.

### RNA isolation and quality control

Total RNA was isolated from frozen tissues (50–100 mg) using TRIzol reagent (Invitrogen, Carlsbad, CA) according to the manufacturer’s instructions. The concentration of total RNA was determined using NanoPhotometer® P330 (IMPLEN, Los Angeles, CA). RNA quality was determined with the Agilent 2100 Bioanalyzer (Agilent Technologies, Massy, France) and only the samples with an RNA integrity number (RIN) >8.5 were further used for RNA-sequencing, and quantitative real-time PCR.

### Library preparation and Illumina sequencing

RNA-Seq libraries were prepared and sequenced at the University of Hawaii Cancer Center Genomics and Bioinformatics Shared Resource (UHCC GBSR) facility. A TruSeq Stranded mRNA kit (Illumina, San Diego, CA) was used to prepare the RNA-Seq libraries from total RNA samples extracted from laying hens at 15–20 h p.o. (n = 3) and non-laying hens (n = 3) uteri. Libraries were prepared according to the manufacturer’s protocol without any modification. Briefly, poly-A RNA fraction, containing mRNAs and certain non-coding RNAs, was enriched from 500 ng of total RNA in two rounds of purification using Poly-T oligo magnetic beads. RNA was eluted using Elute, Prime, Fragment Mix followed by 8-minute fragmentation at 94 °C. Fragmented RNA samples were subsequently used for cDNA first- and second-strand synthesis finished by purification on AMPure XP beads (Beckman Coulter, Brea, CA) that were used to separate the blunt-ended dsDNA from the unincorporated nucleotides and enzymes. cDNAs were then adenylated at their 3′ ends followed by ligation of indexing adapters, specific for each sample. The DNA fragments with adapter molecules on both ends were enriched with 15 cycles of PCR followed by two rounds of purification on AMPure XP beads. The size and quality of the libraries were assessed in a High Sensitivity DNA Bioanalyzer assay (Agilent Technologies, Massy, France). Next, libraries were quantified by qPCR using KAPA Library Quantification Kit (KAPA Biosystems, Boston, Massachusetts) and were normalized to the concentration of 4 nM. Libraries were pooled and denatured using freshly prepared 0.2 N NaOH followed by further dilution with HT1 buffer to obtain a final concentration of 1.8 pM. As a sequencing control, the library pool was spiked-in with 1% (v/v) of 1.8 pM denatured PhiX library and loaded onto the reagent cartridge. The sequencing run was performed with NextSeq500 (Illumina, San Diego, CA), in single-end mode with a read length of 1 × 76 bp. Illumina BaseSpace-created FASTQ files were used for further analysis.

### RNA-sequencing analysis

Single-end reads in FASTQ format were explored using FastQC (Babraham Institute, Cambridge, UK) and cleaned using Prinseq, a perl script^32^. The cleaning procedure included trimming low quality reads from both 3′ and 5′ ends until a base pair of Phred quality score of 30 (99.9% accurate) or greater was found and filtering out reads having a mean quality score less than 30 and length below 30 nucleotides. Cleaned reads were aligned against chicken reference genome Galgal 5.0 using in Array Studio (version10; OmicSoft, Cary, NC^33^). Differential gene expression analysis in layers with respect to non-layers’ groups was performed by the DESeq2 algorithm^34^ as implemented in Array Studio. The genes with at least two-fold change (FC) and Benjamini and Hochberg q-value < 0.05 were called differentially expressed.

### Pathways analyses

The differentially expressed genes (DEGs) were subjected to the Ingenuity Pathway Analysis (IPA; QIAGEN Inc., https://www.qiagenbioinformatics.com/products/ingenuity-pathway-analysis)^35^ to gain insights into the canonical pathways and network discovery. Credible conclusions were derived from the pathways, networks, and functions since the IPA makes the annotation based on the human genome.

### Quantitative real-time RT-PCR (qPCR)

Twelve candidate genes having a predicted function in calcium transport or remodeling were selected for qPCR validation. Primers specific to each gene were designed using NCBI primer blast tool (shown in Supplementary Table S5). The synthesis of first-strand cDNA was performed by reverse transcription of 1 µg total RNA (20 μl reaction of RT mixture) using High-Capacity cDNA Reverse Transcription Kit (Applied Biosystems, Foster City, CA). cDNAs were further diluted with nuclease free-water (1:25) and 3 μl per qPCR reaction was used. Gene expression was analyzed using PowerUp SYBR Green Master Mix (Applied Biosystems, Foster City, CA) on a StepOne Plus real-time PCR system (Applied Biosystems, Foster City, CA). Each 10 μl of PCR reaction mixture consisted of 5 μl PowerUp SYBR Green Master Mix, 1 μl of each forward and reverse primers, and 3 μl of cDNA. PCR reactions were carried out following standard cycling mode. A melting curve was also generated to confirm the sequence-specific PCR products. Three house-keeping genes Glyceraldehyde 3-phosphate dehydrogenase (GAPDH), Beta-actin (B-actin) and TATA-Box Binding Protein (TBP) were analyzed in triplicates in each hen of the experimental groups to determine the most stable house-keeping gene in uterine tissues. The target genes were analyzed in duplicates and expression level was determined using cycle threshold (Ct) values following standard curve method after normalization with TBP. Fold change for each gene was calculated using the 2^−ΔΔCt^ method. Data for fold change were presented as mean ± standard error. Values were subjected to one-way analysis of variance (ANOVA) followed by Tukey-Kramer test for multiple mean comparisons to determine significance at p-value < 0.05 on a SAS platform.

## Electronic supplementary material

Supplementary Dataset 1

## Data Availability

The datasets generated in the current study are available in the Gene Expression Omnibus (GEO) repository and can be accessed with the Accession Number GSE114103.
